# Supplementary material for: Lunar base agent-based modeling - A benchmark for simulating crewed space missions
Source: PLoS One. 2026 May 27;21(5):e0348882. doi: 10.1371/journal.pone.0348882 (PMC13215557; doi:10.1371/journal.pone.0348882)
Supplement: Attachment — Submitted filename: Response to Reviewers.pdf [file pone.0348882.s002.pdf]

Department of Computational and Data Sciences

4400 University Drive, MS 6A12, Fairfax, Virginia 22030
Phone: 703-993-9298 Fax: 703-993-9300
Email: cds@gmu.edu Web: https://cos.gmu.edu/cds

December 14th, 2025
Fairfax, VA

Dear Editors of PLOS One:

Thank you for reviewing our manuscript titled "Lunar base agent-based modeling – A benchmark for simulating crewed space missions" for publication in PLOS One, subject to revisions. We appreciate the time and effort you and the reviewers have dedicated to providing valuable feedback on our work. We have carefully considered all the comments and suggestions provided by the reviewers and have made appropriate revisions to the manuscript. Below, we address each point raised by the reviewers and explain the influence their feedback has had on our new version of the paper.

Academic Editor:

1. Please ensure that your manuscript meets PLOS ONE's style requirements, including those for file naming. The PLOS ONE style templates can be found at: https://journals.plos.org/plosone/s/file?id=wjVg/PLOSOne_formatting_sample_main_body.pdf and https://journals.plos.org/plosone/s/file?id=ba62/PLOSOne_formatting_sample_title_authors_affiliations.pdf

   We used the LaTeX template to create the manuscript at the PLOS One site: https://journals.plos.org/plosone/s/latex. We also verified that the revised manuscript follows the PLOS ONE's style requirements on the websites mentioned above.

2. Please note that PLOS One has specific guidelines on code sharing for submissions in which author-generated code underpins the findings in the manuscript. In these cases, we expect all author-generated code to be made available without restrictions upon publication of the work. Please review our guidelines at: https://journals.plos.org/plosone/s/materials-and-software-sharing#loc-sharing-code and ensure that your code is shared in a way that follows best practice and facilitates reproducibility and reuse.

   We uploaded the code to GitHub: https://github.com/rvera-gmu/Lunar-Base-ABM. We included this in the Data Availability Statement.

3. Please note that funding information should not appear in any section or other areas of your manuscript. We will only publish funding information present in the Funding Statement section of the online submission form. Please remove any funding-related text from the manuscript.

   We removed all funding-related text in the manuscript.

4. Thank you for stating the following financial disclosure:
   "GMU ORIEI Award no. 102264"

Department of Computational and Data Sciences

4400 University Drive, MS 6A12, Fairfax, Virginia 22030
Phone: 703-993-9298 Fax: 703-993-9300
Email: cds@gmu.edu Web: https://cos.gmu.edu/cds

Please state what role the funders took in the study. If the funders had no role, please state: "The funders had no role in study design, data collection and analysis, decision to publish, or preparation of the manuscript."
If this statement is not correct you must amend it as needed.
Please include this amended Role of Funder statement in your cover letter; we will change the online submission form on your behalf.

The funders had no role in study design, data collection and analysis, decision to publish, or preparation of the manuscript. We included this statement in our cover letter.

5. Please expand the acronym "GMU ORIEI" (as indicated in your financial disclosure) so that it states the name of your funders in full.
   This information should be included in your cover letter; we will change the online submission form on your behalf.

   GMU ORIEI (George Mason University Office of Research, Innovation, and Economic Impact). We included this information in our cover letter.

6. Please provide a complete Data Availability Statement in the submission form, ensuring you include all necessary access information or a reason for why you are unable to make your data freely accessible. If your research concerns only data provided within your submission, please write "All data are in the manuscript and/or supporting information files" as your Data Availability Statement.

   All data are in the manuscript and/or supporting information files. This is included in the Data Availability Statement.

7. If the reviewer comments include a recommendation to cite specific previously published works, please review and evaluate these publications to determine whether they are relevant and should be cited. There is no requirement to cite these works unless the editor has indicated otherwise.

   We reviewed all the reviewer comments and cited specific previously published works that were relevant. These specific citations are included in the revised manuscript.

8. After a thorough evaluation, we recognize its value; however, this manuscript requires significant revisions for further consideration. Please enhance the figures' resolution and font size to improve readability, as these figures currently do not meet publication standards.

   We made significant revisions to the manuscript based on all reviewer comments. We updated all figures by enhancing resolution, increasing font size to improve readability, and ensured that they met the publication standards listed in https://journals.plos.org/plosone/s/figures.

Department of Computational and Data Sciences
4400 University Drive, MS 6A12, Fairfax, Virginia 22030
Phone: 703-993-9298 Fax: 703-993-9300
Email: cds@gmu.edu Web: https://cos.gmu.edu/cds

Reviewer #1:

9. I think the model would benefit greatly from considering the data from analogous habitat studies in underwater simulants, cave studies as well as from isolation studies, i.e., performer during the pandemic. Their consideration might help minimize the effects of limited dataset, providing insights not gathered elsewhere. Is there any particular reason you decided not to use such data (10.1016/j.actaastro.2024.10.044, 10.1016/j.actaastro.2021.04.003, 10.1027/1016-9040/a000453)?

   There is relevant TLX data in the DIANA study that we used for model validation and comparison (10.1016/j.actaastro.2024.10.044). Figure 7 and Table 13 were updated to include this additional data point. The other two papers did not have any useful and relevant data for the model or paper. In the Discussion section of the paper, we mentioned that the model can benefit significantly with more related data.

10. Moon communication delay will be an additional stress factor for the crew, additionally increasing the psychological pressure in high-risk situations, when fast decisions are required (~2,5 sec could be even a life-death matter).

    We did not have specific data relating to the impact of communication delay on psychological stress, so it is not explicitly simulated in the ABM. One of the key assumptions on page 8 states that communication systems between Earth and the Moon Base are consistent. We also stated this as a model limitation in the Discussion section that can be explored in future research.

11. Space missions are putting the crew in greater danger than any ground-based habitat: the lack of oxygen in space scenario makes life support system failure even more critical (except for the underwater habitats). Did you consider including the additional "danger factor" for the dataset, so the model would consider the ground-based results as the lower limit of the stress factor associated with the environment?

    The danger perception is similar to what would be experienced in the analogous Antarctic studies, but we do not have additional data to assess the impact of oxygen deficient environments. In the Discussion section, we stated one of the model limitations is the lack of data that is related to coping capacity or stress and analogous environments, which can be used to address this additional factor.

12. You mention the EDEN ISS in your paper, did you consider including the positive effects of in-situ plant production on psychological condition of the crew (10.1007/978-3-030-20503-4_71)?

    The ABM includes ISRU food production. We also included a positive impact to coping capacity based on the quantitative results of the recommended paper (10.1007/978-3-030-20503-4_71).

GEORGE MASON UNIVERSITY

Department of Computational and Data Sciences

4400 University Drive, MS 6A12, Fairfax, Virginia 22030
Phone: 703-993-9298 Fax: 703-993-9300
Email: cds@gmu.edu Web: https://cos.gmu.edu/cds

13. Regarding the areas for your further consideration (not necessarily to be included in the reviewed paper, but for the future model development): The interlink between the psychological and physiological health should not be overlooked by the model. Loss of the crew member is additional factor to impact the psychological health - depending on the reason lowering (well-suited in terms of DISC personalities) or improving (poorly-suited in terms of DISC personalities) during standard crew member change. A separate scenario could be considered fatal loss of a crew member.

    There is a relationship between psychological and physiological health that is not explored by the ABM due to the lack of data availability. We included this as a limitation for future study in the Discussion section. The ABM was also updated to include a negative coping capacity impact due to death of a crew member based on quantitative data collected on grief trajectories in the first months of loss. We ran a separate scenario for fatal loss of a crew member, which can be seen in Case 6 of the Scenario Analysis section.

14. In Author summary section, when mentioning the Agent_Zero framework for the first time, the citation is missing.

    We removed the Author summary section because it is not required for PLOS One submission.

15. L2: The first American orbital space flight took place in 1962 (Mercury-Atlas 6 mission), with astronaut John Glenn onboard. Manned space program in America started in 1961 with 2 suborbital flights within the Mercury program, following the Soviet's Wostok program. In 1986 the first manned flight beyond the Earth's orbit, heading to the Moon, took place during the Apollo 8 mission.

    We rewrote the introduction paragraph to include this information.

16. L7-9: Initially JAXA and CSA were not involved in station building. Maybe rephrasing the sentence would be better, to show both that all the 5 partners together develop ISS today, but the ISS' history started in 1990s.

    We rephrased the sentence to reflect this fact.

17. L80: i in Agent i should be cursive probably. L87, 88: citations to the Latan´e-Darley experiment + social media impact on 2011 Arab Spring uprising needed.

    We made the correction in L80. To simplify the sentence on L87 and L88, we removed the references to the additional studies.

18. L95: "hand-picked by NASA" - I would prefer the phrase "by space agencies," not pointing NASA exclusively. As the Lunar base will finally be used by astronauts of various nationalities (e.g. from European countries), it's worthy to highlight that all the space agencies put equal efforts to prepare their crews for extreme conditions of space missions.

4400 University Drive, MS 6A12, Fairfax, Virginia 22030
Phone: 703-993-9298 Fax: 703-993-9300
Email: cds@gmu.edu Web: https://cos.gmu.edu/cds

We changed the sentence to include, “by space agencies.”

19. L192-194: source of information on Perseverance power source and its lifetime needed.

    We included the source of information for the Perseverance power source in the References section: NASA Jet Propulsion Laboratory. Mars 2020 Perseverance Launch Press Kit.

20. L202: Gateway will not be on the Moon but on Moon's orbit, please try to rephrase the sentence ("(...) a theoretical habitat environment on the Moon (...)")

    We rephrased the sentence to: "The main objective of the model is to simulate a theoretical lunar mission environment, including both the surface habitat (Moon Base) and the orbiting Gateway station, for astronauts to perform relevant space mission tasks."

21. Table 2, physical-health attribute: does the value degrade over time? The impact of lowered/microgravity on muscle mass, bone density etc. on humans should affect the parameter additionally over time.

    The baseline duration for the Lunar Base ABM is three months. The long duration physical impact on astronauts is included as a model limitation in the Discussion section.

22. Personality_type attribute: what about all the in-between DISC personality types?

    There is no data to support the model for more detailed personality types and interactions. This is included as a model limitation in the Discussion section.

23. Table 4, unexpected_task_prob attribute: where from comes the starting value? Could you provide some references here?

    Due to the lack of data on the probability of unexpected activity, we changed this parameter using a uniform random variable (0,1) for the initial case. See Case 3 in the Results section for the model impact due to unexpected_task_prob.

24. Table 6, shipment of resources: Does the probability of shuttle accident reflect the probability of not getting the resupply and is included in the model?

    Yes. Probability of not getting the resupply is included in the ABM.

25. Table 9: Is the number of hours of sleep fixed permanently or can it be shortened by e.g. unexpected accident? Can the simulation include the necessity to work past normal time and how it affects Astronaut's health (e.g. by slowing down the recovery) ?

    The Lunar Base ABM was updated to include a sleep deprivation probability and stress impact from historical ISS data due to unexpected activity or personal lack of sleep.

GEORGE MASON UNIVERSITY

Department of Computational and Data Sciences

4400 University Drive, MS 6A12, Fairfax, Virginia 22030
Phone: 703-993-9298 Fax: 703-993-9300
Email: cds@gmu.edu Web: https://cos.gmu.edu/cds

According to the ISS data, if astronauts sleep less than 6 hours, they experience an increase of stress, which will negatively impact their coping capacity.

26. L333-338: Are the mentioned changes reflected by changes in Crew stress levels?

    These changes are not reflected in the Crew stress levels due to the lack of data availability. In the Discussion section of the paper, we mentioned that the model can benefit significantly with more related data.

27. L451: The model is generally well-described, but I encourage you to share the code publicly (e.g. via GitHub), to increase its visibility and chances for reuse/results reproduction: 10.1016/j.envsoft.2020.104873.

    We uploaded the code to GitHub: https://github.com/rvera-gmu/Lunar-Base-ABM and included this link in our Data Availability Statement.

28. L599-607: This limitation should be expressed more clearly. For now, the weakest point of the model as tension level is strictly related to interpersonal dynamics which the model tries to explore and define its dependencies with other stressors. Establishing the rationale of such model's behaviour should be described in more detail, maybe consider even creating a separate chapter where all the relevant aspects could be discussed.

    We updated the tension model to more accurately represent the increase in frustration or tension an astronaut will feel based on the TLX test. According to the visual test for the NASA-TLX methodology, there are 20 ticks (or divisions) for each of the dimensions in the TLX score with a rating scale from 0 to 100. Consequently, a meaningful or significant increase is 1 tick in the rating scale that equates to 100/20 = 5 or a 0.05 increase on a scale from 0 to 1. This incremental change aligns more closely with the results obtained by the Antarctic studies and is shown in Figure 6 in the Results section. Previously, we were using a 0.01 increase, and a 0.05 increase is more representative of a meaningful or significant frustration level increase.

29. L644: CL - I assume the abbreviation stands for Confidence Levels? Please add full name with the first abbreviation appearance.

    We updated Tables 15-18 to show mean, standard deviation, minimum, maximum, and median summary statistics instead of confidence levels (CLs) to make the results and analysis clearer.

30. Discussion section: Please consider to broaden the section, especially to point out the potential data sources for the model improvement (so not only the analogue habitats and Antarctica studies but also all the rest mentioned above). Put it in the context of how such data translates to space conditions and what kind of studies would be needed to improve the model.

4400 University Drive, MS 6A12, Fairfax, Virginia 22030
Phone: 703-993-9298 Fax: 703-993-9300
Email: cds@gmu.edu Web: https://cos.gmu.edu/cds

In the revised expanded Discussion section of the paper, we stated this as a model limitation and stated that the model can benefit significantly with more related data. We mentioned what kind of studies would be needed to improve the model.

31. Conclusions section: Include the current model limitations, especially the interpersonal tension level.

    The updated Discussion section of the paper includes the current model limitations and recommendations for future research.

32. References: Reference list in over 1/3 are websites - please add the missing citation where applicable and maybe try to provide additional references wherever possible for the Internet ones?

    We added five additional non-internet references or academic papers that were not included in the original manuscript.

33. Figure 1, Incidental variables: typo, should probably be environmental changes (a is missing).

    We updated Figure 1 to be more readable and understandable and verified that there are no typographical errors.

Reviewer #2:

34. I suggest refining the purpose of the paper. It reads heavily like a white paper explaining the methods to design the ABM but also discusses some basic results of the model. I suggest making it clearer to readers that this is a methodology paper and provide proof of validity of the model and publish the results separately or significantly reducing the methodology of how the model was built and focus more on the output/results of the model. This is a strong paper regardless of the final direction.

    We wanted to show how we can use ABMs for space missions. The main goal of the paper is to model future space missions and point to red flags related to human factors for mission failure. This is a benchmark paper that can be used for both modeling space missions and for phenomena that are data poor for future events. The methodological approach is a result of the research for this benchmark model and paper.

35. Methods and materials: Authors define the Lunar Base as a complex system twice, this is not necessary. The models are used for both human and system-wide inputs. The setup of this section is somewhat confusing. I suggest restructuring, and using less parenthetical commentary, such as, "…as a matter of fact."

    In the Materials and methods section, the Artemis program was defined as a complex system and the Lunar Base as a complex adaptive system because we were trying to make the distinction that the latter includes adaptations to exogenous and endogenous factors.

GEORGE MASON UNIVERSITY

Department of Computational and Data Sciences

4400 University Drive, MS 6A12, Fairfax, Virginia 22030
Phone: 703-993-9298 Fax: 703-993-9300
Email: cds@gmu.edu Web: https://cos.gmu.edu/cds

We removed the second reference to the Lunar Base as a complex system and deleted the phrase, "as a matter of fact."

36. Depending on the audience you want to attract, it may be worthwhile to put the application of the Agent_Zero model and the fight or flight response into a diagram to allow those who are unfamiliar to get a better understanding. Also consider further describing the 2011 Arab Spring uprising- as readers may not be familiar with it or 'affective homophily.' Consider moving the limitations of Epstein's model to the conclusions and limitations. Define the Rescorla-Wagner rule.

    For simplification and clarity, we removed the references to the 2011 Arab Spring and defined homophily. We left the limitations of the Epstein's model in this section because it does not significantly impact the Lunar Base ABM. Agent_Zero was used only to inspire the integration of the cognitive, emotional, and social aspects of the Lunar Base ABM. The underlying mechanics of each component (and their interactions) are significantly different from Epstein's model.

37. Figures 2 & 3 are too blurry to read or interpret. All the graphs and figures are blurry, but those two are unreadable.

    We modified Figure 2 by increasing font size and only showing major attributes and functions by removing counters, calculated fields, and unnecessary attributes. For Figure 3, we increased the font, modified the text for clarity/readability, and removed unnecessary parameters. We updated all figures by enhancing resolution, increasing font size to improve readability, and ensured that they met the publication standards listed in https://journals.plos.org/plosone/s/figures.

38. Table 9- please confirm the schedule, is this theoretical, or based on the literature? I do not think that the time dedicated to exercise has been officially determined.

    Table 9 is based on a NASA fact sheet and historical ISS schedules. We included additional citations in the References section. Overall, the daily schedule contains an 8-hour sleep cycle, 2 hours of exercise, daily mission control conferences/planning, routine maintenance and cleaning tasks, and at least 6 hours of EVA operations. This schedule is a representation of daily astronaut activity and can be modified for different missions.

39. Results and discussion: Remove figures 4-6 and the discussion around them regarding the normal distributions to visualize the data or run the analyses again with the data and their true distributions. It is not reasonable to display these and then utilize them as results. This reads as poor scientific methodology, whereas authors clearly put immense intention into building the model.

    These figures (now referred to as Figures 5 to 7) are updated by removing the normal distributions on the analogous data and replaced with horizontal error bars (mean, standard deviation) in order to visually compare the ranges between the ABM output and the

4400 University Drive, MS 6A12, Fairfax, Virginia 22030
Phone: 703-993-9298 Fax: 703-993-9300
Email: cds@gmu.edu Web: https://cos.gmu.edu/cds

historical data. We also included Tables 11 to 13 for the summary statistics data of the relevant figures.

40. The discussion is far too short. Please expand. Put limitations of the model in this section instead of the methods. If keeping results in the paper, add how this information can be applied to future missions, etc.

    We expanded the Discussion section to include model limitations, analysis on model results, recommendations for future missions, and opportunities for future research.

Reviewer #3:

41. It would greatly benefit the manuscript if the authors revisited the order in which material is presented. On first read, the first half of the manuscript is very difficult to follow and does not provide the context needed to understand what the authors set out to investigate. On page 3, it is still not clear whether the ABM would be predominantly focused on factors related to the 'lunar environment' or 'human factors' (or both, or something else). It is not until around the discussion of the design concept (page 14) that the context is made clear.

    On page 3 (Materials and methods section), we state: "In our model, astronauts and autonomous rovers, each with individual preferences, strategies, and behaviors, represent agents. The ABM environment reflects the lunar geography, resources, and other factors (e.g., gravity, temperature, radiation, solar wind, regolith, moonquakes, and impact gardening) that can limit or promote agent interactions." This is a concise description of the ABM components that is explained further in subsequent pages.

42. Here are two specific examples where context would help, but the authors should go through the entire paper for other cases as well.
    Page 2 "The lunar environment also encompasses endogenous and exogenous factors… …reinforced by positive and negative feedback loops…" This finally made more sense on page 13 after the reference to Figure 3.
    Page 3 "…heterogenous actors and the flow of information, energy, and resources."

    Pages 2 and 3 are meant to explain why the lunar base mission is a complex system and how ABMs are well suited to study and explore their dynamics. The subsequent pages build upon this idea that culminates in the development of a fully functional ABM that can be used to simulate the complexity of a lunar base environment and astronaut team dynamics.

43. The manuscript leading up to the design concept section is somewhat vague and perhaps assumes the reader already understands the approach and components of the ABM. For instance, the attributes of the agents are described between pages 3 and 6, before their importance to the ABM is understood. The 'agents' are finally introduced in the middle of a paragraph on page 7. After reading the design concept section, it was much easier to go back and understand the pages leading up to it.

GEORGE MASON UNIVERSITY

Department of Computational and Data Sciences

4400 University Drive, MS 6A12, Fairfax, Virginia 22030
Phone: 703-993-9298 Fax: 703-993-9300
Email: cds@gmu.edu Web: https://cos.gmu.edu/cds

Pages 3 to 6 provide the necessary background for the significant components of the ABM. On pages 7 to 13, we describe the design concepts, assumptions, model description, ABM class diagram, agent attributes, and scheduler. Pages 13 to 16 provides a description of the model output and provides an input-output flow diagram (Figure 3) that summarizes the previous pages.

44. The use of terms with specific vs. generic meanings should be reduced throughout the manuscript as much as possible unless the context can be made clear. i.e. terms such as “skills”, “attributes”, “tasks”, ‘activities’, ‘endogenous’, ‘exogenous’ ‘parameters’, ‘variables’, ‘components’, ‘factors’, ‘interactions.’ Also check for consistency, for example: ‘Moon Base’ vs ‘Lunar Base’.

Skills refer to professional skill requirements necessary to successfully perform mission tasks (Table 10). Attributes are the specific characteristics of an agent (Tables 2 to 8). Tasks are the specific activity performed by the agent according to the scheduler (Table 9). Activities refer to astronaut tasks and other functions to include sleeping, physical exercise, personal times, etc. Exogenous and endogenous parameters are shown in Figure 3. Variables, components, and factors are synonymous with the ABM parameters. Interactions refer specifically to the agent-to-agent and agent-to-environment interactions of an ABM. The "Moon Base" refers to the specific agent in the model. "Lunar Base" refers to the entire ABM as a whole.

45. Tables 2 through 8. These tables are very helpful in providing context. The column labeled ‘Starting Value (or range)’ needs some clarification. The ranges are more helpful than only providing a starting value. It is understood (or assumed), for instance, that “base_waste” in Table 4 should only be positive values, but is there a max value associated with this attribute or does a missing range mean there is no upper limit? Also in Table 4: Starting location is set to ‘Moon’. What is the reason for including this attribute; are other locations possible in this case?

We updated the column to state: "Starting Value(s)". If it shows a single number, then the ABM starts at that value. For example, base_waste's starting value is 0.0. It can increase according to the model dynamics. If the starting value is a range, then the ABM starts with a random value between that range. For example, eva_skill starting value(s) is 0.0-1.0. This means that the ABM can start with a random number between this range (e.g., 0.532) that explains the difficulty requirement to perform this skill. We removed the location attribute because it is only used in the task assignment algorithm. The Moon Base can only be located on the Moon.

46. Page 12: “Together, these four components…” Not clear to which four components it refers.

We rewrote the sentence to: "Together, these four key components (i.e., professional skills, coping capacity, tension level, and efficiency rate) offer a basis for simulating astronaut behavior under extreme environmental conditions."

Department of Computational and Data Sciences
4400 University Drive, MS 6A12, Fairfax, Virginia 22030
Phone: 703-993-9298 Fax: 703-993-9300
Email: cds@gmu.edu Web: https://cos.gmu.edu/cds

47. The fonts in Figures 2 and 3 are too small to read in standard letter format.

    We modified Figure 2 by increasing font size and only show major attributes and functions by removing counters, calculated fields, and unnecessary attributes. For Figure 3, we increased the font, modified the text for clarity/readability, and removed unnecessary parameters. We updated all figures by enhancing resolution, increasing font size to improve readability, and ensured that they met the publication standards listed in https://journals.plos.org/plosone/s/figures.

48. The beginning of the results section seems to be more of a method description. More details should be provided about the different cases that were. Table 11 provides some info, but it is insufficient to discern what parameters are the same vs. different between each of the cases. For instance, how many astronauts in cases 2-5. What are the locations in cases 2-5. This table could be replaced with a table that provides the relevant parameters consistently for all cases.

    Table 14 (Lunar Base ABM initial and scenario cases) distinctly and sufficiently describes each case. The initial scenario is the starting point for all other cases. The description of all the other cases explains the difference compared to the initial case. For example, Case 5 contains all the starting values of the initial case (i.e. 4 astronauts, 2 rovers, 3-month mission, baseline values) and the only change is the total mission duration increases to 6 months. If we included a table of all the parameters for every scenario, it would be very large and cause additional confusion over the excessive detail.

49. The final subsections within the results may be more suitable for the discussion. These interpretations could be further expanded. For instance, based on this model, do the authors conclude anything regarding optimum crew sizes, transfer rates, mission duration, tension reduction, etc.? It may be premature to extrapolate beyond the limits of this model, but it could still make for an interesting discussion.

    We expanded the Discussion section to include model limitations, analysis on model results, recommendations for future missions, and opportunities for future research.

Reviewer #4:

50. Page 2, line 25: "While engineering and technology innovation drives space missions,..." - This affirmation is subjective. What drives space exploration can be many things. Please rephrase or remove. The rest of the phrase is fine. I suggest: "While engineering and technology innovation is necessary for space missions, understanding human and operational dynamics is crucial for mission success. Even more so if the objective is to establish long term presence on the Moon."

    We made the recommended changes.

GEORGE MASON UNIVERSITY

Department of Computational and Data Sciences

4400 University Drive, MS 6A12, Fairfax, Virginia 22030
Phone: 703-993-9298 Fax: 703-993-9300
Email: cds@gmu.edu Web: https://cos.gmu.edu/cds

51. Page 2, line 34, The authors mention NASA's human factors and behavioral research. Is there a reference(s) to this research? I would like to know which specific research is this model based on. This is also necessary for verification and reproduction of the method.

    We added 2 additional citations for this sentence relating to NASA papers on teamwork in long duration space missions and the NASA Human Research Program (HRP).

52. Page 3, line 49. This phrase has several explanations inside parenthesis () which are expendable because it does not add essential information and adds reading complexity. I suggest to remove them along with their content. Besides, the phrase that follows explains what are the bottom-up and -down layers.

    We removed this phrase from the sentence.

Reviewer #5

53. It has to be accepted that this type of work has many different parameters (participants' physiological, cognitive, psychological, social and similar etc.) that should be considered but difficult to integrate with small number of participants. Thus, maybe a larger dataset should be used to evaluate such a study here, but this is very rare to find that number of participants due to ethical situations and volunteer groups. Even if the authors do not have such option, this is understandable, of course.

    We updated the paper to include all data that was used in the study. This can be found in Tables 11 to 18. The Lunar Base ABM is meant to be a useful space mission planning tool rather than a theoretical methodology study that can be improved with more data. We added the model limitations of different parameters and lack of data in the Discussion section.

54. I strongly suggest authors integrate much more explanatory diagrams/charts for what they are presenting. Rather than boxes and interactions/connections

    We significantly modified Figures 1 to 3 (i.e., concept diagram, ABM class diagram, and input-output flow model) to improve clarity on the model dynamics, agents, parameters, and interactions. Tables 2 to 8 have all the details behind the agent attributes and Table 9 explains the ABM scheduler or list of daily activities.

55. Data presented in Figs. 2 and 3 are not readable.

    We modified Figure 2 by increasing font size and only show major attributes and functions by removing counters, calculated fields, and unnecessary attributes. For Figure 3, we increased font size, modified the text for clarity/readability, and removed unnecessary parameters. We updated all figures by enhancing resolution, increasing font size to improve readability, and ensured that they met the publication standards listed in https://journals.plos.org/plosone/s/figures.

4400 University Drive, MS 6A12, Fairfax, Virginia 22030
Phone: 703-993-9298 Fax: 703-993-9300
Email: cds@gmu.edu Web: https://cos.gmu.edu/cds

56. Conclusion is short and understandable. However, it would be much better to enhance this section a bit more to give the idea and the importance of the effort spent.

    We expanded the Discussion section instead of the Conclusion section to include model limitations, analysis of model results, recommendations for future missions, and opportunities for future research.

57. If possible, readers would want to see the Python codes to reproduce the results and/or develop the code.

    We uploaded the code to GitHub: https://github.com/rvera-gmu/Lunar-Base-ABM. We included the link in the Data Availability Statement.

Reviewer #6:

58. The paper presents a strong and well-structured ABM, but the introduction could better emphasize what makes this model unique. A short paragraph contrasting it with previous Mars or lunar simulations and explaining how it integrates psychological variables such as coping and tension would make its novelty clearer.

    We believe that the Lunar Base ABM is a novel concept compared to any previous space simulation because it integrates human behaviors, interactions, and environmental factors at the astronaut level that will be present in the future Artemis missions that would be difficult to compare to other models. While prior research in space psychology and NASA Task Load Index (TLX) has advanced understanding of human performance in isolated and confined environments, to our knowledge this is the first study to integrate these elements into an ABM specifically for lunar missions, enabling direct scenario testing for future Artemis operations. The Lunar Base ABM is meant to be an effective benchmark tool that can enhance future lunar exploration, which is discussed in the Introduction section. We left the explanation of the integration of the psychological variables in the Materials and methods section.

59. The adaptation of Epstein's Agent Zero is a creative foundation, though the mechanics need clarification. It would help to specify how the cognitive, affective, and social parameters were scaled, how often they update, and what range of thresholds (τ) was tested. Since the logic is multiplicative rather than additive, a short equation or pseudo-code would make the implementation easier to follow.

    The description of task thresholds and teamwork needs more detail. Readers should know how the model aggregates multi-skill cooperation, whether individual skills are added, averaged, or multiplied, and what specific threshold values were used for task success.

    We uploaded the code to GitHub: https://github.com/rvera-gmu/Lunar-Base-ABM. This explains all the details of how the ABM was implemented. Equation 3, which is a modified version of Equation 1 (i.e., Agent_Zero model) explains how the ABM uses the cognitive, affective, and social parameters and is further elaborated in the code. The cognitive or

Department of Computational and Data Sciences
4400 University Drive, MS 6A12, Fairfax, Virginia 22030
Phone: 703-993-9298 Fax: 703-993-9300
Email: cds@gmu.edu Web: https://cos.gmu.edu/cds

professional skills are randomly generated from 0 to 1. The effective or emotional component consists of randomly generated values of coping capacity and tension (equally weighted). As stated in the Materials and methods section, the astronaut dispositions are modeled as multiplicative instead of additive because the nonlinear structure captures the compounding effects of psychological health degradation under stress and professional skill learning improvement over time considering personality traits and environmental factors. In contrast, the Agent_Zero model is additive with the cognitive and affective components. Nonlinearity is introduced through the Rescorla-Wagner effect of the emotional factor. The Lunar Base ABM task thresholds are also randomly assigned from 0 to 1. Teamwork involves a pair of astronauts working together by adding their total disposition (Equation 3) to exceed the randomly assigned task threshold. Specific tasks required specific skills as shown in Table 10. The GitHub code provides all the underlying mechanics of the Lunar Base ABM.

60. The DISC-based personality reactivity coefficients (D = 0.3, C = 0.4, S = 0.5, I = 0.7) seem reasonable but appear judgment-based. A brief justification or sensitivity check would confirm robustness. Clarify whether these penalties occur once per event or accumulate over time.

These coefficients were judgment-based. In the revised manuscript and updated model. We removed them and now the emotional component is based on a simple average between coping capacity and tension.

61. The learning-curve formulation is well motivated but incomplete. Please indicate which slope values were actually applied to each task category, whether learning occurs at the individual or team level, and whether it affects time-to-complete or probability of success.

The learning curve or efficiency rate is applied in Equation 3 and affects probability of success at the team level. In the paragraph before Equation 3, we updated the statement: "The learning rate is initialized based on NASA benchmarks with a typical slope of 85%. This experience factor increases the effective disposition of tasks compounding the cognitive and emotional variables over time, resulting in an overall probability increase of task success at the team level." In the Modeling Agent_Astronaut psychology and performance section, we state: “The benefits of the technology or experience learning curve are only applicable to repetitive and labor-intensive activities, such as assembly, maintenance, and mission planning.” Lastly, Table 4 description for the initial scenario includes "0.85 learning rate".

62. The NASA TLX integration is one of the paper’s strengths but remains under-explained. It would help to map TLX subscales (mental, physical, temporal, effort, frustration, performance) to model variables such as task complexity or coping capacity and to specify if TLX is updated per task or per day and how subscales are weighted. Explicitly linking Figure 1 to these components would turn it into a real part of the model rather than a reference diagram.

Department of Computational and Data Sciences
4400 University Drive, MS 6A12, Fairfax, Virginia 22030
Phone: 703-993-9298 Fax: 703-993-9300
Email: cds@gmu.edu Web: https://cos.gmu.edu/cds

We updated Figure 1 to show how the TLX subcomponents (i.e., mental demand, physical demand, temporal demand, performance, effort, and frustration) are integrated in the conceptual framework.

63. Stochastic parameters should include their full distributions and time bases. The reported moonquake probability (0.00096 day^{-1}) seems low and might need a magnitude qualifier, while the shuttle accident rate (0.06456) should be clarified as per-flight, not per-day. These details are essential for reproducibility.

These daily probabilities are explained in the Setting parameters section, and the implementation of these environmental probabilities can be found in the GitHub code. We also updated Tables 4 to 6 to identify if these events are daily probabilities or per-flight probabilities.

64. The model's handling of emergency events could be clearer. A brief explanation of whether astronauts abort ongoing EVAs or finish the current hour before reacting would make the resilience logic more transparent.

Unexpected or emergency events occur randomly throughout the day. If the astronaut encounter one of these events, they will stop what they are doing and perform troubleshooting. According to the code, for each hour, the ABM will check for unexpected events first before proceeding with the daily activities.

65. Resource and energy relationships deserve one line of clarification, whether energy limits task execution or if resource production draws power from the system. If not yet modeled, noting that its planned would prevent confusion.

In the discussion section, we state: "model can be expanded to analyze energy and resource production and consumption." Although, the ABM includes the mechanism for tracking energy and resource production (i.e., astronauts need resources to survive and the Moon Base needs energy to operate), we didn't explicitly study the results for this paper. This is a topic for future studies and is mentioned in the Discussion section.

66. Because the study aims to provide a benchmark, the authors should consider including the code or at least a full parameter appendix listing every stochastic input, range, and time unit so others can replicate the results.

We uploaded the code to GitHub: https://github.com/rvera-gmu/Lunar-Base-ABM. This is included in the Data Availability Statement.

67. In the results, the baseline case reports a 20% task completion rate, but breaking down critical versus non-critical tasks would show whether mission performance risks are hidden within aggregated totals.

GEORGE MASON UNIVERSITY

Department of Computational and Data Sciences

4400 University Drive, MS 6A12, Fairfax, Virginia 22030
Phone: 703-993-9298 Fax: 703-993-9300
Email: cds@gmu.edu Web: https://cos.gmu.edu/cds

We included the statement following the 20% task completion rate sentence: "This productivity rate does not include the completion of unexpected tasks because they are outside the baseline of daily scheduled mission activities."

68. The histograms fitted with normal curves are useful visuals, but since ABM outputs are rarely normal, it would be better to report medians and interquartile ranges or note that the normal fits are illustrative only.

We updated these figures (Figures 5 to 7) by removing the normal distributions on the analogous data and replaced them with horizontal error bars (mean, standard deviation) in order to visually compare the ranges between the ABM output and the historical data. Also, we included Tables 11 to 13 for the actual summary statistics data of the relevant figures.

69. Coping aligns well with Antarctic analogs, but tension is unrealistically low. Acknowledge that the baseline excludes strong social stressors (no transfers, stable schedules) and that conservative reactivity settings likely suppress tension variability.

We updated the tension model to more accurately represent the increase in frustration or tension an astronaut will feel based on the TLX test. According to the visual test for the NASA-TLX methodology, there are 20 ticks (or divisions) for each of the dimensions in the TLX score with a rating scale from 0 to 100. Consequently, a meaningful or significant increase is 1 tick in the rating scale that equates to 100/20 = 5 or a 0.05 increase on a scale from 0 to 1. This incremental change aligns more closely with the results obtained by the Antarctic studies and is shown in Figure 6 in the Results section. Previously, we were using a 0.01 increase, and a 0.05 increase is more representative of a meaningful or significant frustration level increase.

70. The TLX calibration using Equation 4 is clear but should restate that it only rescales outputs and does not alter simulation dynamics. Clarify what the threshold value (≈67.7) actually represents, an empirical percentile or an acceptable workload limit.

The results section states: "The calibration methodology in Equation 4 was used to adjust the synthetic TLX score to align it with the EDEN ISS study ([29], [30]), which is the most analogous data to use for further analysis. The adjustment only alters the interpretation of the TLX values to examine the impact of input parameter changes without affecting the fundamental simulation dynamics. The simulated range demonstrates that most synthetic TLX scores fall within the middle to upper range of empirical benchmarks, suggesting moderate to high workload and mission effort." Also, we altered the statement to: "there is an 85% chance that TLX scores will remain below 72.55, a threshold consistent with the acceptable workload limit during analog missions."

71. In the scenario analysis, a brief mechanism summary would sharpen interpretation: Case 1's larger crew improves specialization and compatibility; Case 2's frequent transfers disrupt cohesion; Case 3's extra tasks raise stress; Case 4's higher learning slope weakens efficiency;

Department of Computational and Data Sciences
4400 University Drive, MS 6A12, Fairfax, Virginia 22030
Phone: 703-993-9298 Fax: 703-993-9300
Email: cds@gmu.edu Web: https://cos.gmu.edu/cds

Case 5's extended mission increases fatigue. Showing monthly trends for Case 5 would reveal how stress accumulates.

For clarity and conciseness, the significant ABM results are summarized in the following sections: "Total task results", "Coping capacity results", "Tension level results", "Synthetic TLX score results." We decided to present the results this way because not every scenario (i.e., Cases 1 to 9) significantly impacts the output parameters.

72. Along with percentage changes, include absolute deltas and standardized TLX shifts to make comparisons across scenarios clearer.

We updated Table 18 to include absolute values and % deltas compared to the initial case for the mean values.

73. The discussion would benefit from connecting findings to actionable insights, crew sizing as an efficiency lever, transfer cadence as a stability factor, and training investment to offset steep learning slopes, and from acknowledging the optimistic baseline assumptions that reduce tension and risk.

We expanded the Discussion section to include model limitations, analysis on model results, recommendations for future missions, and opportunities for future research.

We hope that these revisions have significantly improved the quality and clarity of our manuscript. We have uploaded the revised version, the version with track-changes, along with this rebuttal letter, better resolution images, and updated Data Availability Statement to include the GitHub code link. We look forward to your feedback on the revised manuscript and would be happy to address any further questions or concerns you may have. Thank you for your consideration.

Sincerely,

Anamaria Berea, PhD
Associate Professor
Computational and Data Sciences
Director of Graduate Studies
Computational Social Sciences
Department of Computational and Data Sciences
College of Science
MAIS Concentration Head for CSS
College of Humanities and Social Sciences
George Mason University
ph: 703-993-3624
Research Hall
Room 226, MSN 6A12
Fairfax, VA, 22030
